# Randomness, Informational Entropy, and Volatility Interdependencies among the Major World Markets: The Role of the COVID-19 Pandemic

**DOI:** 10.3390/e22080833

**Published:** 2020-07-30

**Authors:** Salim Lahmiri, Stelios Bekiros

**Affiliations:** 1 Department of Supply Chain & Business Technology Management, John Molson School of Business, Concordia University, Montreal, QC H3H 0A1, Canada; salim.lahmiri@concordia.ca; 2 Department of Economics, European University Institute, 50014 Florence, Italy; 3 Rimini Centre for Economic Analysis, Wilfrid Laurier University, 75 University Ave W., Waterloo, ON N2L 3C5, Canada

**Keywords:** COVID-19 pandemic, Bitcoin, stock market, precious metal market, energy market, GARCH, wavelet packet Shannon entropy, hierarchical clustering

## Abstract

The main purpose of our paper is to evaluate the impact of the COVID-19 pandemic on randomness in volatility series of world major markets and to examine its effect on their interconnections. The data set includes equity (Bitcoin and Standard and Poor’s 500), precious metals (Gold and Silver), and energy markets (West Texas Instruments, Brent, and Gas). The generalized autoregressive conditional heteroskedasticity model is applied to the return series. The wavelet packet Shannon entropy is calculated from the estimated volatility series to assess randomness. Hierarchical clustering is employed to examine interconnections between volatilities. We found that (*i*) randomness in volatility of the S&P500 and in the volatility of precious metals were the most affected by the COVID-19 pandemic, while (*ii*) randomness in energy markets was less affected by the pandemic than equity and precious metal markets. Additionally, (*iii*) we showed an apparent emergence of three volatility clusters: precious metals (Gold and Silver), energy (Brent and Gas), and Bitcoin and WTI, and (*iv*) the S&P500 volatility represents a unique cluster, while (*v*) the S&P500 market volatility was not connected to the volatility of Bitcoin, energy, and precious metal markets before the pandemic. Moreover, (*vi*) the S&P500 market volatility became connected to volatility in energy markets and volatility in Bitcoin during the pandemic, and (*vii*) the volatility in precious metals is less connected to volatility in energy markets and to volatility in Bitcoin market during the pandemic. It is concluded that (*i*) investors may diversify their portfolios across single constituents of clusters, (*ii*) investing in energy markets during the pandemic period is appealing because of lower randomness in their respective volatilities, and that (*iii*) constructing a diversified portfolio would not be challenging as clustering structures are fairly stable across periods.

## 1. Introduction

Since the outbreak of the COVID-19 pandemic, various studies were conducted to investigate its effect on the economy, including its impact on the correlations between crude oil and agricultural futures [1], co-movement between COVID-19 and Bitcoin [2], the tourism industry [3], the covariance between temperature, COVID-19 and exchange rate in Wuhan [4], Italian manufacturing firms [5], B2B sales forces [6], efficiency of equity and cryptocurrency markets [7], airline employment [8], entrepreneurial uncertainty [9], consumer behavior [10], marketing innovations [11], corporate social responsibility and marketing [12], and randomness and mutual information between markets [13], to name few. In addition, other interesting studies focused on the forecasting of new cases [14] and knowledge sharing and collaboration for preparedness to fight the pandemics [15].

The main purpose of the current study is (*i*) to evaluate the volatility randomness in Bitcoin, Standard and Poor’s 500 (S&P500), Gold, Silver, West Texas Instruments (WTI), Brent, and Gas market, (*ii*) to investigate the interdependence structure of volatilities in these markets, and (*iii*) to provide an overview of the dynamic interrelationships among the volatility series by considering the co-movement within two sub-periods. Specifically, we distinguish between the period prior to the COVID-19 pandemic and period during the occurrence of the pandemic.

Our motivation for measuring randomness in volatility series is to evaluate the irregularity in information content in such series. Indeed, volatility series are good proxy of investor expectations and perceived risk. Hence, it is interesting to measure randomness in volatility series to assess the effect of the COVID-19 on regularity in investor’s expectations and perceived risk. The motivation for analyzing the volatility co-movements between Bitcoin, S&P500, Gold, Silver, WTI, Brent, and Gas, is twofold. First, all these markets are heavily subject to speculation and market expectations. Second, they are representative of cryptocurrency, stock, precious metal, and energy major markets. Third, revealing connectedness of world major markets helps summarizing and visualizing their associations, a fact that provides investors useful information about the linkages between various major markets for better investment decisions.

The methodology employed uses the generalized autoregressive conditional heteroskedasticity (GARCH) model [16] to estimate volatility series, wavelet packet Shannon entropy (WPSE) [17,18] to measure randomness in estimated volatility series, and hierarchical clustering [19,20] to identify the natural structure of the set composed of volatility series and eventually disclose the composition of the clusters. The GARCH-based volatility estimation model is the simplest and most parsimonious standard stochastic process to estimate time series volatility, while the wavelet packet-based entropy has a prevailing capability to embody the intrinsic characteristics of the original data in all frequency bandwidths. The hierarchical clustering algorithm is a methodology that robustly explores the clustering of a dataset to mine this information for connectedness visualization. It is worth nothing that GARCH-based models, entropy, and hierarchical clustering have successfully been applied to model volatility [21,22,23,24,25,26,27,28], to evaluate randomness in financial and economic data [29,30,31,32,33,34,35], and to cluster financial data [36,37,38,39,40,41].

In sum, our study contributes to the recent existing literature in four important ways. Firstly, we investigate the impact of the COVID-19 pandemic on world major markets; namely cryptocurrency, equity, precious metal, and energy markets. Indeed, these four categories of markets attract most of the investors worldwide. Secondly, the study identifies markets with increased randomness in volatility due to the pandemic. Thirdly, we attempt to shed light on the new formed clusters due to the pandemic. Fourthly, the obtained results are expected to show markets with less irregularity and low level in volatility and potential portfolio diversification opportunities.

The rest of the paper is organized as follows. Section 2 briefly describes the methodology applied to estimate the results. Section 3 presents the data and provides the empirical results. The main conclusions are set out in Section 4.

## 2. Methods

### 2.1. The GARCH Process

The GARCH model [16] is the most popular volatility model thanks to its flexibility and ability to capture volatility clustering. Let consider the return time series (*r_t_*) of an asset at time *t* be defined as first difference of logarithmic prices. Then, the standard GARCH(*p*,*q*) model of a return time series can be described as follows:

(1) $$r_{t}=\mu_{t}+\epsilon_{t}=\mu_{t}+h_{t}\eta_{t}$$

(2) $$h_{t}^{2}=\omega +\sum_{i=1}^{p}\alpha_{i}h_{t-i}^{2}+\sum_{j=1}^{q}\beta_{j}\varepsilon_{t-j}^{2}$$

where $\eta_{t}∼iid\left(0,1\right)$, *μ_t_* denotes the conditional mean and *h_t_* is the conditional variance with the sufficient conditions *ω* > 0, *α* ≥ 0, *β* ≥ 0 to ensure *h_t_* > 0. Accordingly, *h_t_* is the estimated volatility of return series *r_t_*. The appropriate orders *p* and *q* are determined based on Akaike information criterion.

### 2.2. The Wavelet Packet Shannon Entropy

Shannon entropy [17] offers a valuable benchmark for evaluating and comparing a probability distribution. Indeed, it affords a measure of information randomness of any signal. In this study, Shannon entropy is calculated in wavelet packet transform domain. In fact, the wavelet packet transform is a generalization of the standard wavelet transform as it produces the full decomposition of the original signal. Therefore, this attractive feature allows the wavelet packet transform to not suffer a loss of information with respect to the original signal. The wavelet packet transform of the original time series *s*(*t*) is recursively computed as follows [42]:

(3) $$\{\begin{array}{c} d_{0,0}\left(t\right)=s\left(t\right)\\ d_{i,2j-1}\left(t\right)=\sqrt{2}\sum_{k}h\left(k\right)d_{i-1,j}\left(2t-k\right)\\ d_{i,2j}\left(t\right)=\sqrt{2}\sum_{k}g\left(k\right)d_{i-1,j}\left(2t-k\right) \end{array}$$

where *h*(*k*) is a high-pass filter, *g*(*k*) is a low-pass filter, and *d_i,j_* are the coefficients of the wavelet packet transform at the *i*th level for the *j*th node. Then, the wavelet energy *E_i,j,k_* can be used to measure the information of the *k*th coefficient as follows:

(4) $$E_{i,j,k}=\left\|d_{i,j,k}\right\|{}^{2}$$

Next, the total energy is expressed as follows:

(5) $$E_{i,j}=\sum_{k=1}^{N}E_{i,j,k}$$

where *N* denoted the number of the coefficients in the node. Subsequently, the probability of the *k*th coefficient is given as follows:

(6) $$p_{i,j,k}=\frac{E_{i,j,k}}{E_{i,j}}$$

where

(7) $$\sum_{k}p_{i,j,k}=1$$

Finally, the wavelet packet Shannon entropy (WPSE) is calculated as follows:

(8) $$SE_{i,j}=-\sum_{k=1}^{N}p_{i,j,k}\times log\left(p_{i,j,k}\right)$$

### 2.3. Hierarchical Clustering

Hierarchical clustering allows organizing multivariate data into clusters with respect to homogeneities among the data. Hence, the obtained clusters have similar components that are close to each other and distant from other components in the other clusters. As a result, the partitioning enhances the understanding of the data by disclosing its internal structure. The correlation distance between two different volatility times *S_p_* and *S_q_* is given by:

(9) $$corr\left(S_{p},S_{q}\right)=1-\frac{\left(S_{p}-{\bar{S}}_{p}\right){\left(S_{q}-{\bar{S}}_{q}\right)}^{T}}{\Sigma }$$

where $\bar{S}$ represents the average of *S*, *T* denotes the transpose operator, and Σ is expressed as follows:

(10) $$\Sigma ={\left(\left(S_{p}-{\bar{S}}_{p}\right){\left(S_{q}-{\bar{S}}_{q}\right)}^{T}\right)}^{1/2}{\left(\left(S_{p}-{\bar{S}}_{p}\right){\left(S_{q}-{\bar{S}}_{q}\right)}^{T}\right)}^{1/2}$$

Then, the Ward’s linkage denoted by *d*(*S*_1_, *S*_2_) is used to locate the distance between two clusters *S*_1_ and *S*_2_ as follows:

(11) $$d\left(S_{1},S_{2}\right)=\sqrt{\frac{2n_{1}n_{2}}{n_{1}+n_{2}}}\left\|\bar{c_{1}}-\bar{c_{2}}\right\|$$

where *n* is the number of samples and $\bar{c}$ is the centroid of a cluster.

## 3. Data and Results

We collected daily closing prices from three distinct group markets: equity, precious metals, and energy. The equity group includes Bitcoin and S&P500, the precious metals group includes Gold and Silver, and the energy group includes West Texas Instruments (WTI), Brent, and Gas markets. The pre-pandemic period spans from 1 August 2019 to 31 December 2019 and the pandemic period from 2 January 2020 to 26 May 2020. For each market, we computed the return series as the first log-differences in prices. Then, volatility series of return series are estimated by using the GARCH model. Subsequently, for each market, the wavelet packet Shannon entropy is computed from its corresponding volatility series. Finally, hierarchical clustering is applied to the set composed of all estimated volatility series to obtain the connection structure between them.

**Figure 1:**
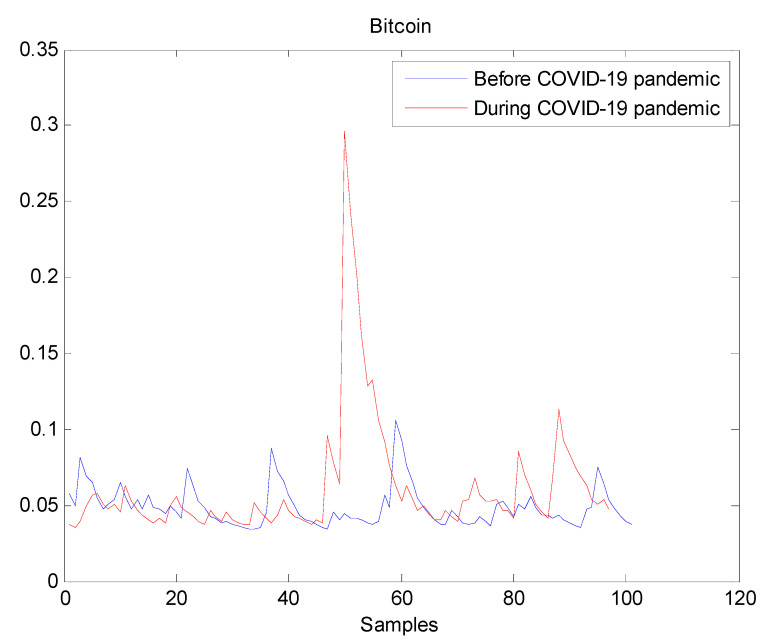
Plot of volatility $\left(h_{t}^{2}\right)$ dynamics of Bitcoin market. The pre-pandemic period spans 1 August 2019 to 31 December 2019 and the pandemic period from 2 January 2020 to 26 May 2020. There are several peaks in Bitcoin volatility during the pandemic. This suggests that the COVID-19 pandemic seriously affected Bitcoin volatility.

**Figure 2:**
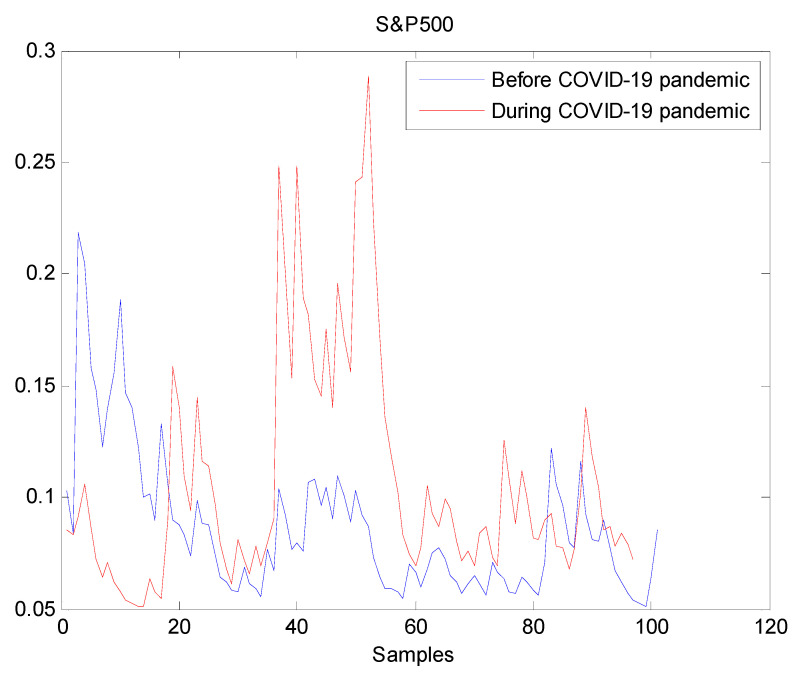
Plot of volatility $\left(h_{t}^{2}\right)$ dynamics of S&P500 market. The pre-pandemic period spans 1 August 2019 to 31 December 2019 and the pandemic period from 2 January 2020 to 26 May 2020. There are many large peaks in S&P500 volatility during the pandemic. This suggests that the COVID-19 pandemic seriously affected S&P500 volatility.

**Figure 3:**
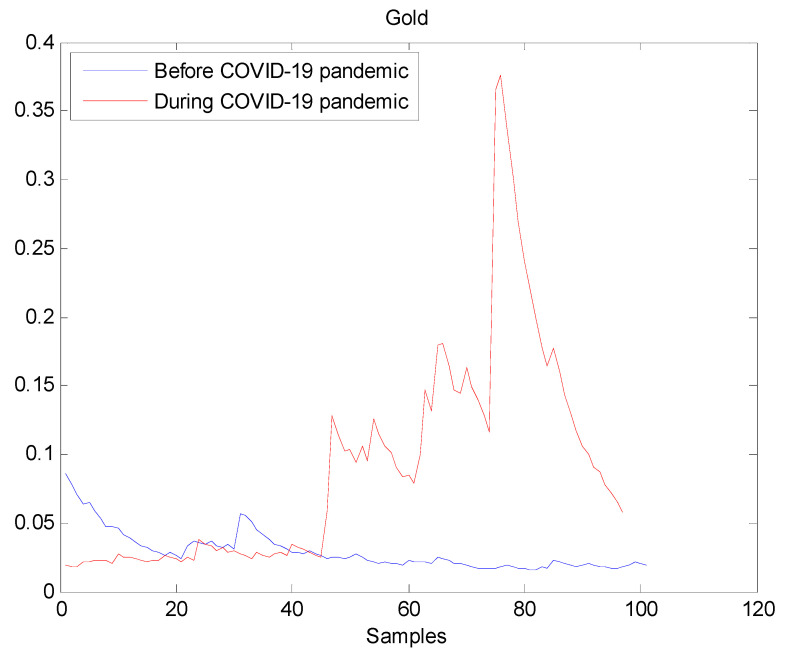
Plot of volatility $\left(h_{t}^{2}\right)$ dynamics of Gold market. The pre-pandemic period spans 1 August 2019 to 31 December 2019 and the pandemic period from 2 January 2020 to 26 May 2020. The behavior of Gold volatility is smooth during the pre- pandemic period. On the contrary, there are many large peaks in Gold volatility during the pandemic. This fact suggests that the COVID-19 pandemic seriously affected Gold volatility.

**Figure 4:**
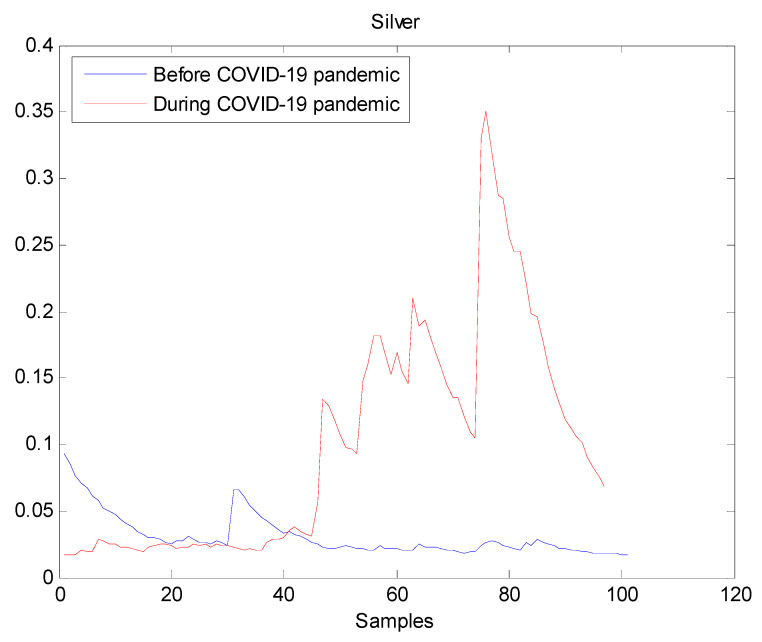
Plot of volatility $\left(h_{t}^{2}\right)$ dynamics of Silver market. The pre-pandemic period spans 1 August 2019 to 31 December 2019 and the pandemic period from 2 January 2020 to 26 May 2020. The behavior of Silver volatility is smooth during the pre- pandemic period. On the contrary, there are many large peaks in Silver volatility during the pandemic. Therefore, the COVID-19 pandemic seriously affected Silver volatility.

**Figure 5:**
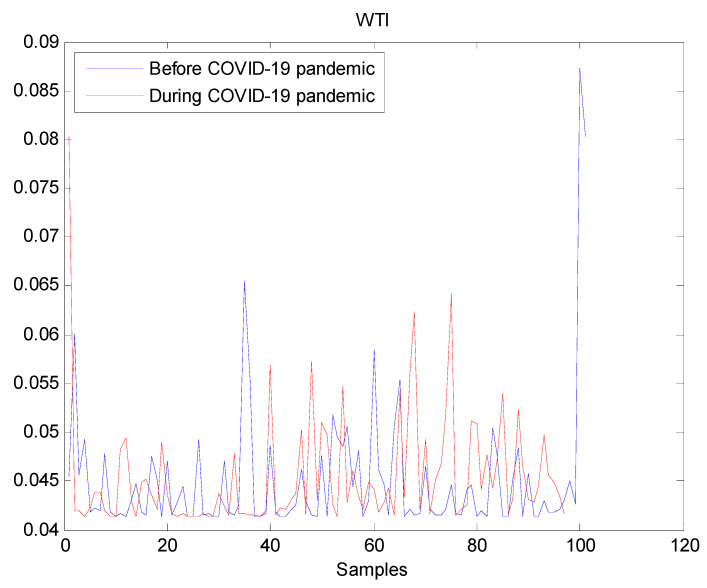
Plot of volatility $\left(h_{t}^{2}\right)$ dynamics of WTI market. The pre-pandemic period spans 1 August 2019 to 31 December 2019 and the pandemic period from 2 January 2020 to 26 May 2020. WTI volatility series before and during the pandemic look similar which suggests that the pandemic has not significantly affected WTI volatility.

**Figure 6:**
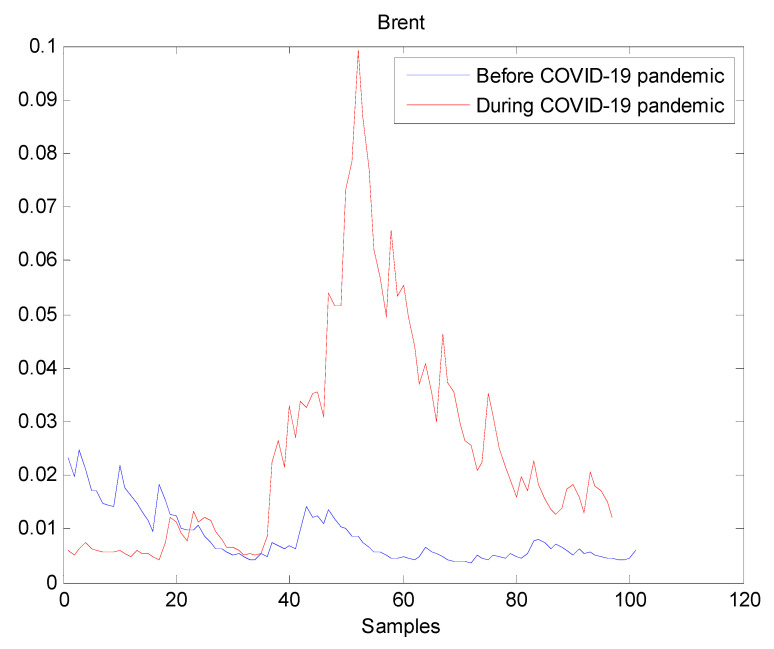
Plot of volatility $\left(h_{t}^{2}\right)$ dynamics of Brent market. The pre-pandemic period spans 1 August 2019 to 31 December 2019 and the pandemic period from 2 January 2020 to 26 May 2020. The behavior of Brent volatility is decreasing during the pre- pandemic period. However, there are many large peaks in Brent volatility during the pandemic. It indicates that Brent volatility has increased during the pandemic, thereby the COVID-19 pandemic seriously affected Brent volatility.

**Figure 7:**
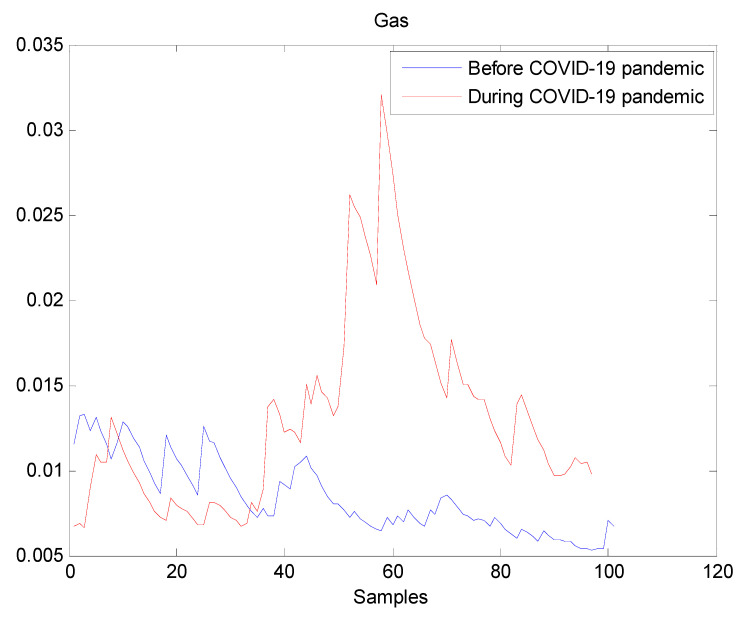
Plot of volatility $\left(h_{t}^{2}\right)$ dynamics of Gas market. The pre-pandemic period spans 1 August 2019 to 31 December 2019 and the pandemic period from 2 January 2020 to 26 May 2020. The behavior of Gas volatility is decreasing during the pre- pandemic period. However, there are many large peaks in Gas volatility during the pandemic period. It indicates that Gas volatility has increased during the pandemic, which may suggest that the COVID-19 pandemic seriously affected Gas volatility.

The estimated volatilities are shown in Figure 1, Figure 2, Figure 3, Figure 4, Figure 5, Figure 6 and Figure 7 respectively for Bitcoin, S&P500, Gold, Silver, WTI, Brent, and Gas market. Accordingly, it is observed that the level of volatility $\left(h_{t}^{2}\right)$ dynamics during the COVID-19 pandemic is more pronounced than that in the pre-pandemic period. In addition, there are clear and strong different peaks in the level of volatility $\left(h_{t}^{2}\right)$ during the pandemic compared to the pre-pandemic one. One plausible explanation is that these peaks are most likely linked to response of investors to shocks due to the pandemic. For instance, the peaks may refer to a contraction which represents a phase of the business cycle whereby the entire market is in decline. Then, the sudden and strong increase in volatility is apparently linked to market uncertainty associated with the COVID-19 pandemic.

Besides, one may observe that volatility is different between the pre and during pandemic period. To formally check if differences exist between across periods for each market, the one-side *t*-test and one-side *F*-test are applied to check respectively if (*i*) the average volatility in pre-pandemic period is lower than the average volatility during the pandemic period, and if (*ii*) the variability in volatility in pre-pandemic period is lower than the variability in volatility during the pandemic period. The computed probability values (*p*-values) are reported in Table 1 for each single market.

According to the computed *p*-values from *t*-test shown in Table 1, the null hypothesis that the average volatility before the COVID-19 pandemic is less than that during the pandemic is strongly rejected for all markets, except for WTI market. Therefore, the average volatility series in Bitcoin, S&P500, Gold, Silver, Brent, and Gas market have not increased during the COVID-19 pandemic. Indeed, it decreased. However, the average volatility series in WTI has increased during the pandemic period. Besides, based on the computed *p*-values from *F*-test shown in Table 1, the null hypothesis that volatility variability before the COVID-19 pandemic is less than that during the pandemic is strongly rejected for all markets, except for WTI market. Therefore, the variability in volatility series of Bitcoin, S&P500, Gold, Silver, Brent, and Gas market has statistically decreased during the COVID-19 pandemic. Though, the variability in volatility series of WTI has significantly intensified during the pandemic period.

In short, the results from statistical tests provided in Table 1 shows that during the COVID-19 pandemic, average volatility and variability in volatility has decreased in all markets, except in WTI market. In this regard, one could observe that the volatility in oil markets follows two distinct behaviors. For instance, the volatility in WTI market increased during the pandemic, while the volatility of the Brent market decreased.

**Figure 8:**
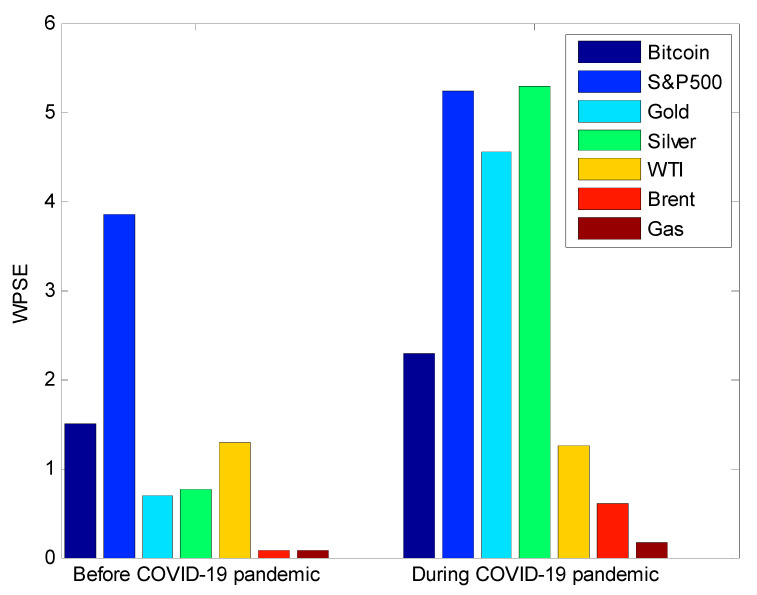
Wavelet packet Shannon entropy (WPSE) prior and during the COVID-19 pandemic. As depicted, WPSE has significantly increased during the COVID-19 pandemic in all markets. For the WTI market, WPSE remained similar across the two periods.

Figure 8 shows the estimated values of the wavelet packet Shannon entropy (WPSE) prior and during the COVID-19 pandemic. Accordingly, its value significantly increased during the COVID-19 pandemic with respect to Bitcoin, S&P500, Gold, Silver, Brent, and Gas markets. Specifically, there was a strong increase in precious metal markets. In short, the information randomness levels in S&P500 stock market volatility and precious metal market volatilities were the most affected by the COVID-19 pandemic. Besides, the value of the wavelet packet Shannon entropy in WTI volatility has slightly decreased during the COVID-19 pandemic.

Also, according to Figure 8, volatilities in Brent and Gas markets are the only one showing the lowest wavelet packet Shannon entropy before and during the pandemic compared to the remaining volatilities. More specifically, it is observed that all energy markets embedded the lowest randomness level in volatility information content during the pandemic.

**Figure 9:**
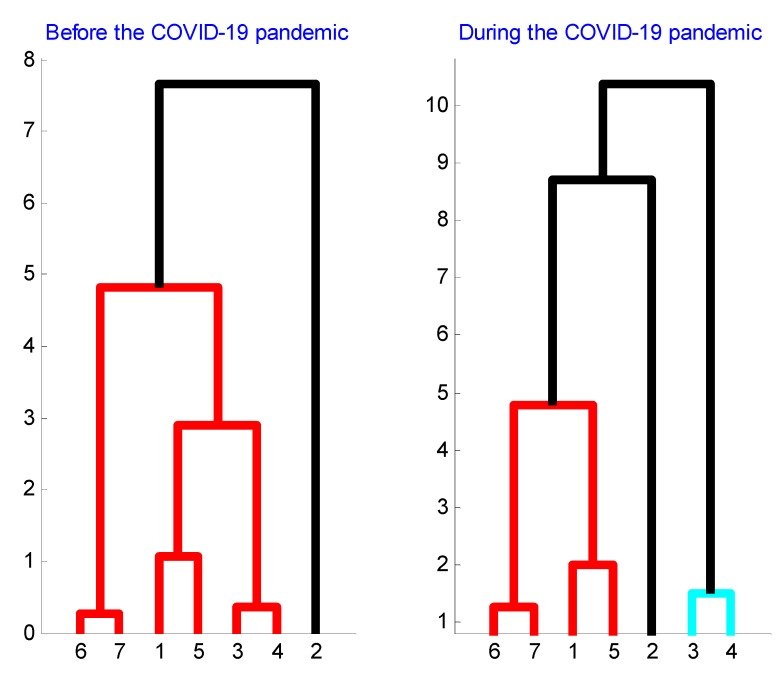
Clustering of volatility co-movements between markets before and during the COVID-19 pandemic. The clustered markets are Bitcoin (1), S&P500 (2), Gold (3), Silver (4), WTI (5), Brent (6), and Gas (7). The connection between Brent and Gas, between Bitcoin and WTI, and between Gold and Silver is clear and stable through periods. The S&P500 forms a unique cluster before the pandemic. During the pandemic, Gold and Silver form a unique cluster and the S&P500 appears connected to Brent and Gas cluster, and to Bitcoin and WTI cluster. The COVID-19 has clearly affected the interconnections between world large markets.

Finally, according to Figure 9, there are three clear volatility clusters formed before and during the COVID-19 pandemic: Brent and Gas, Bitcoin and WTI, and Gold and Silver. Hence, the constituents of each volatility cluster evolve through each period in a similar way. In other words, the structure of each volatility cluster has not been changed due to the pandemic which suggests that volatility cluster constituents are strongly related to each other. Besides, it is shown that S&P500 market volatility was less connected to all three main volatility clusters in the period before the pandemic. However, during the pandemic period, the S&P500 market volatility became connected to the cluster formed by Bitcoin and WTI market volatility and to the cluster formed by Brent and Gas market volatility. In addition, during the pandemic period, the cluster grouping volatilities of Gold and Silver market became disconnected from the other two major volatility clusters (Brent and Gas cluster and Bitcoin and WTI cluster).

In short, our empirical findings can be summarized as follows:

- The randomness in volatility of S&P500 and randomness in volatility of precious metals were the most affected by the COVID-19 pandemic.
- The randomness in energy markets was less affected by the COVID-19 pandemic than equity and precious metal markets.
- There is clear emergence of three volatility clusters: precious metals (Gold and Silver), energy (Brent and Gas), and Bitcoin and WTI. The S&P500 volatility is a unique cluster.
- In the period prior to the pandemic, the S&P500 market volatility was not connected to volatilities of Bitcoin, energy, and precious metal markets.
- During the pandemic, the S&P500 market volatility became connected to volatility in energy markets and volatility in Bitcoin.
- During the pandemic, volatility in precious metals is less connected to volatility in energy markets and to volatility in Bitcoin market.

## 4. Conclusions

In this study, we examined the effect of the COVID-19 pandemic on information randomness in volatility of seven world major markets represented in three distinct groups: equity, precious metals, and energy markets. In addition, we analyzed the connection structure between volatility series prior and during the pandemic. We found that the level of randomness measured by wavelet packet Shannon entropy has increased during the COVID-19 pandemic. Additionally, our hierarchical clustering results showed that in both periods there exists a close co-movement between volatilities of Gold and Silver, between volatilities of Brent and Gas, and between volatilities of Bitcoin and WTI. Furthermore, before the pandemic, the S&P500 volatility represented a single and isolated cluster. During the pandemic, the S&P500 volatility was connected to energy cluster and to the cluster formed by Bitcoin and WTI. Moreover, we found that Bitcoin volatility and S&P500 volatility are not forming a single distinctive cluster. Therefore, they were not directly connected before and after the COVID-19 pandemic.

Yet, another interesting finding is that the volatility in WTI market has increased during the pandemic, while the volatility of the Brent market has decreased. This could be explained by the fact that the Russia-Saudi Arabia oil price war of 2020 has negatively affected oil producers in Texas, but not oil producers in Northern Europe.

Our empirical findings have significant implications for international investors. For instance, the co-movements between precious metal markets volatilities (Gold and Silver) and between energy markets volatilities (Brent and DAS) before and during the COVID-19 pandemic suggest that an international investor would not be able to obtain simultaneously the benefits of portfolio diversification and time diversification. Specifically, during both periods, potential for portfolio diversification does not exist between (*i*) Gold and Silver, (*ii*) between Brent and DAS, and (*iii*) Bitcoin and WTI. However, an investor may diversify his portfolio across single constituents of clusters by investing in Gold, Brent, and Bitcoin, for instance. In addition, investing in energy markets during the pandemic period is appealing because of lower randomness in their respective volatilities. Furthermore, constructing a diversified portfolio would not be challenging since clustering structures are more or less stable.

## Figures and Tables

**Table 1:** Reported *p*-values from *t*-test and *F*-test.

	*t*-Test	*F*-Test
	Null Hypothesis	Null Hypothesis
	Average Volatility before Pandemic < Average Volatility during Pandemic	Volatility Variability before Pandemic < Volatility Variability during Pandemic
**Bitcoin**	0.0018	1.5883 × 10^−24^
**S&P500**	3.6033 × 10^−04^	6.9059 × 10^−06^
**Gold**	1.8539 × 10^−12^	2.3640 × 10^−48^
**Silver**	1.5720 × 10^−13^	1.5845 × 10^−46^
**WTI**	0.4042	0.9488
**Brent**	8.6506 × 10^−13^	6.0961 × 10^−37^
**Gas**	7.2314 × 10^−13^	4.8083 × 10^−19^

All statistical tests are performed at 5% statistical significance level. A *p*-value less than 5% yields to a rejection of the null hypothesis.
